# Avoidance of Healthcare Utilization in South Korea during the Coronavirus Disease 2019 (COVID-19) Pandemic

**DOI:** 10.3390/ijerph18084363

**Published:** 2021-04-20

**Authors:** Minjung Lee, Myoungsoon You

**Affiliations:** 1 Department of Public Health Sciences, Graduate School of Public Health, Seoul National University, Seoul 08826, Korea; leciel84@snu.ac.kr; 2 Office of Dental Education, School of Dentistry, Seoul National University, Seoul 08826, Korea; 3 Institute of Health and Environment, Seoul National University, Seoul 08826, Korea

**Keywords:** coronavirus, COVID-19, SARS-CoV-2, healthcare utilization, healthcare avoidance, public health

## Abstract

Avoidance of healthcare utilization among the general population during pandemic outbreaks has been observed and it can lead to a negative impact on population health. The object of this study is to examine the influence of socio-demographic and health-related factors on the avoidance of healthcare utilization during the global outbreak of a novel coronavirus (COVID-19) in 2020. Data were collected through an online survey four weeks after the Korea Centers for Disease Control and Prevention (KCDC) confirmed the first case in South Korea; 1000 subjects were included in the analysis. The logit model for regression was used to analyze the associations between sociodemographic and health-related factors regarding the avoidance of healthcare utilization. Among the participants, 73.2% avoided healthcare utilization, and there was no significant difference in the prevalence of healthcare avoidance between groups with (72.0%) and without (74.9%) an underlying disease. Sociodemographic characteristics (e.g., gender, age, income level, and residential area) were related to healthcare avoidance. Among the investigated influencing factors, residential areas highly affected by COVID-19 (i.e., Daegu/Gyeoungbuk region) had the most significant effect on healthcare avoidance. This study found a high prevalence of healthcare avoidance among the general population who under-utilized healthcare resources during the COVID-19 outbreak. However, the results reveal that not all societal groups share the burden of healthcare avoidance equally, with it disproportionately affecting those with certain sociodemographic characteristics. This study can inform healthcare under-utilization patterns during emerging infectious disease outbreaks and provide information to public health emergency management for implementing strategies necessary to improve the preparedness of the healthcare system.

## 1. Introduction

The outbreak of a novel coronavirus (COVID-19), first appeared in Wuhan [1,2] and has been a major public health threat worldwide. On 20 January, South Korea confirmed its first case [3], and an explosive increase in the number of COVID-19 patients appeared in late February in Daegu city, contributed by a religious group called Shincheonji [4]. In this time, South Korea was one of the hardest hit areas during the global outbreak of COVID-19. As the number of confirmed cases rapidly increased, the Korean government raised the alert level from orange to red on 23 February 2020, and mandated school closures [4]. As of 28 March 2020, the number of COVID-19 cases in Korea reached 9478, including 144 deaths. Among the public, the perceived risk of COVID-19 infection increased and became pervasive; widespread postponing or canceling of social events, avoiding crowded places, and reducing the use of public transportation, subsequently occurred [5].

Decrease in healthcare utilization occur frequently during pandemic outbreaks. In Korea, medical utilization (both admissions and outpatient treatments) decreased during the MERS epidemic in June and July of 2015 when compared to the numbers from 2014 and 2013 [6], along with visits to the emergency department [7]. The overall use of healthcare decreased by 18% during the peak of the Ebola Virus Disease (EVD) outbreak in West Africa [8,9]. Likewise, significant reductions in ambulatory care (23.9%), inpatient care (35.2%), and dental care (16.7%) were observed in Taiwan during the peak of the Severe Acute Respiratory Syndrome (SARS) outbreak [10]. However, a decrease in healthcare utilization patterns during outbreaks can adversely affect population health. Failure to access preventive and urgent life-saving treatments alike can lead to additional deaths [9], and the severity of illness or chronicity of disease, functional or physical disability, and even mortality, are predictable outcomes [11]. Previous studies have suggested that changes in healthcare utilization patterns due to public health emergencies have increased mortality rates from infectious disease as well as non-infectious diseases [12,13,14]. Moreover, increased expenditures related to healthcare as delayed diagnosis as well as more costly multimodal treatments, might be required. As a result, the burden associated with decreases in healthcare utilization may reduce the overall efficacy of a healthcare system.

Healthcare utilization may have declined for several reasons. First, the outbreak may have affected the supply of health due to closures of some health facilities during outbreaks. For example, in Korea, a 35-year-old man employed at a hospital developed symptoms of COVID-19 on February 2. He transmitted it to several patients between 2 and 17 February before he was discharged from his job. The hospital subsequently closed, and 14 additional confirmed cases from this hospital had been reported as of 2 March 2020 [4]. As another example, some hospitals were forced to close as some patients did not properly describe their symptoms of COVID-19 due to concerns of not being admitted and treated [15]. In West Africa, health workers experienced a particularly heavy death toll; many healthcare workers had died, and the supply of healthcare was affected during the EVD outbreak [9].

Second, the demand for healthcare might also have changed. Avoiding visits to healthcare facilities even when sick, or healthcare avoidance behavior, can negatively affect the population’s well-being [11]. It might impede positive health-seeking behaviors and delay care, lead to non-adherence with treatment regimens, or result in a total lack of access to the healthcare system. According to a study in Korea, 34.5% of respondents reported that they avoided hospital visits even when they were ill during the MERS outbreak [16]. Potential patients may have avoided seeking care at health facilities because they feared contracting an infectious disease if they visited during outbreaks. Several studies have reported that concerns about the potential for nosocomial transmission of the disease led to beliefs that health facilities should be avoided. For instance, a Taiwanese study showed that the public’s fears of SARS strongly influenced access to care [10] and a study of Hong Kong residents in the initial stage of the H1N1 outbreak reported that 63.4% of respondents avoided visiting hospitals due to perceived high risk [17].

The potentially severe impact of COVID-19 outbreak on people’s access to healthcare is an important area of study. A critical challenge is to determine how healthcare agencies should respond to changes in healthcare utilization and possible barriers to access healthcare facilities for the public created by the COVID-19 outbreak. Moreover, the lessons learned from the MERS experience in Korea [6] and other countries demonstrate the importance of understanding the community response [17,18,19,20,21,22,23]. To our knowledge, no other study has evaluated the impact of COVID-19 on the demand of healthcare utilization among the general population. In this study, we focus on the avoidance of healthcare utilization or changes in healthcare-seeking behaviors of the public during the COVID-19 outbreak. The aims of the study are two-fold. First, we examine the prevalence of healthcare avoidance among the general population during the COVID-19 outbreak. Second, we investigate the factors associated with healthcare avoidance and identify the vulnerable populations. The results of this study can inform healthcare utilization patterns during infectious disease outbreaks and understanding the factors which affect the access of timely care will inform public health emergency management for implementing strategies necessary to improve the preparedness of the healthcare system.

## 2. Materials and Methods

### 2.1. Study Design

We adopted a cross-sectional survey design to evaluate the public’s avoidance of healthcare utilization during the COVID-19 epidemic using an anonymous online questionnaire. The survey was conducted via an online platform from a research company called Korea Research. The company recruited respondents by sending survey invitations containing general information about the survey, such as its aim and consent statement via e-mail or text message, to registered survey panel members who met the inclusion criteria. The inclusion criteria were as follows: (1) aged 18 years or older, (2) a resident in South Korea, and (3) a Korean speaker. The company sampled respondents using age, sex, and a geographic region-based proportional and quota sampling process. The respondents provided electronic informed consent which appeared on the first page of the survey, and the company protects the confidentiality of anonymous respondents. The target sample size was 999, determined by identifying the smallest acceptable size of a demographic subgroup with a ±3.1% margin of error and a confidence level of 95% [24,25].

Over 1000 subjects completed the surveys, and 1000 were included in the analysis after excluding incomplete responses. The data collection took place over three days (25–27 March), two months after the Korea Centers for Disease Control and Prevention (KCDC) confirmed the first case at the early stage of the epidemic and just before 10,000 cases had been reported (3 April).

### 2.2. Measurements

The outcome variable was the avoidance of healthcare utilization, which respondents self-reported. Respondents self-reported the frequency of the action—“I avoided visiting hospitals even when I was sick”—they have taken during the previous week using a 4-point Likert-type scale (never, sometimes, often, and always). To conduct a logistic regression analysis, we converted the responses into binary answers (never = 0 and otherwise = 1).

Independent variables were categorized into two groups: sociodemographic and health-related factors. Sociodemographic factors included gender (1 = male, 2 = female), age, family size (i.e., living alone, more than 2 persons), marital status (i.e., married, single, divorced, bereaved), and the presence of children younger than elementary school at home (yes = 1, none = 0). We also assessed the education level (1 = middle school or below to 3 = college and above) and the monthly household income in Korean won (KRW) (1 = 200 million KRW or below to 4 = 600 million KRW or above). We collected information about the respondents’ residences (urban = 1, rural = 2) and residential areas, including Seoul, Incheon/Gyeonggi, Daejeon/Sejong/Chungcheong, Gwangju/Jeolla, Daegu/Gyeongbuk, Busan/Ulsan/Gyeongnam, and Gangwon/Jeju regions. The occupation status included whether the respondent was a salary earner, self-employed, or if the respondent was unemployed.

Subjective health status (very poor = 1, poor = 2, moderate = 3, good = 4, Excellent = 5) was investigated to assess health-related factors. To conduct a logistic regression analysis, we converted the responses into ternary answers (poor = 1, moderate = 2, good = 3). We also investigated the presence of underlying disease (e.g., hypertension, dyslipidemia, diabetes, chronic cardiac disease, asthma, and cancer, and others) (Table 1).

### 2.3. Statistical Analysis

We conducted statistical analyses using R version 3.5.1 (R Foundation for Statistical Computing, Vienna, Austria). All the results of quantitative variables were reported by mean (M), standard deviation (SD), or frequency (%) (Table 1). To determine the role of sociodemographic and health-related factors on healthcare utilization avoidance, differences in socio-demographics and health-related factors were compared with the healthcare utilization avoidance using the chi-square statistics (Table 2). The logit model for regression analyzed the associations between sociodemographic factors (e.g., gender, age, family size, education, marital status, income, and employment) and health-related factors (i.e., subjective health and presence of underlying disease) toward one’s avoidance of healthcare utilization. Confounding factors were explored by comparing the differences between the adjusted odds ratio (aOR) in multivariate analysis and the crude odds ratio (OR) in a bivariate analysis of each independent variable on healthcare utilization avoidance (Table 3). Additionally, to examine the moderating effect of gender and the presence of an underlying disease, the same logit model for regression was performed among subgroup participants along with gender (Table 4) and the presence of underlying disease (Table 5).

## 3. Results

### 3.1. Sociodemographic and Health-Related Characteristics

Among the 1000 respondents, there were 478 men (47.8%) and 522 women (52.2%), with a mean age of 47.04 years (M = 47.04, SD = 15.04) (Table 1). The majority of respondents had a family size of more than two persons (90.1%), and 64.9% were married. Half of the respondents had at least some college education (49.0%), followed by those with only a high school education (48.1%). The most common monthly household income was approximately 2.00–3.99 million KRW ($1688–$3369; 31.5%), followed by over 6.00 million KRW ($5065; 29.4%) and 4.00–5.99 million KRW ($3377–$5057; 26.2%) (Table 1). Among the respondents, 88.0% lived in urban areas, and about 9.7% had young children in the home. Regarding occupation status, 47.3% were salary earners, 39.6% were unemployed, and 13.1% were self-employed or held other jobs.

### 3.2. Avoidance of Healthcare Utilization

**Figure 1:**
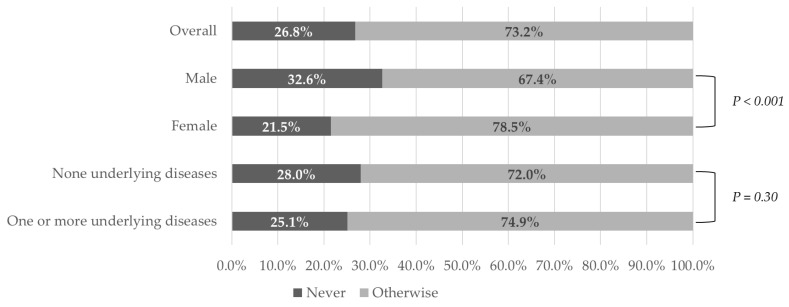
Healthcare utilization avoidance among subgroup participants based on gender and the presence of an underlying disease.

Among the respondents, 26.8% reported that they never avoided visiting hospitals when they were sick (Figure 1). However, 26.6% reported that they did sometimes, 22.3% often, and 24.3% reported that they “always” avoided healthcare utilization when they were unwell. Table 2 reports the Chi-square statistics for variables related to the avoidance of healthcare utilization and describes the group differences in avoidance behavior. Women (*p* < 0.001) and married respondents (*p* = 0.02) were more likely to avoid healthcare. Group differences among age (*p* < 0.001) and residential area (*p* = 0.01) were statistically significant. Among the residential areas, respondents in the Daegu/Gyeongbuk region reported the highest rate of healthcare avoidance (84.8%). However, group differences between respondents with more than one or no underlying disease were not statistically significant (Figure 1).

### 3.3. Factors Influencing the Avoidance of Healthcare Utilization

We used logit regression models to test the association between the avoidance of healthcare utilization and respondents’ sociodemographic factors and health-related factors (Table 3). Out of the sociodemographic factors, female sex (odds ratio (OR), 1.91; 95% confidence interval (CI), 1.40–2.62; *p* < 0.001), age in 50 s (OR, 1.93; 95% CI, 1.06–3.50; *p* = 0.03) and living in rural area (OR, 0.65; 95% CI, 0.41–0.99; *p* = 0.05) were significant individual predictors of healthcare avoidance. Among residential areas, respondents who live in the Daegu/Gyeongbuk region (OR, 3.10; 95% CI, 1.62–5.94; *p* < 0.001), Gangwon/Jeju (OR, 2.78; 95% CI, 1.12–6.88; *p* = 0.03) and Daejeon/Sejong/Chungcheong-do (OR, 2.04; 95% CI, 1.14–3.65; *p* = 0.02) were more likely to practice avoidance than those living in Seoul, the capital city of South Korea. Interestingly, none of the health-related factors were associated significantly with the dependent variable. Respondents who are women in their 50s living in urban and residential areas (especially the Daegu/Gyeongbuk region) are vulnerable in healthcare utilization.

Table 4 and Table 5 provide the results of the subgroup analysis, which show a moderate effect of gender and presence of underlying disease. Among men (*n* = 478), socio-demographic factors such as monthly household income level 4.00–5.99 million KRW (OR = 0.43; 95% CI, 0.18–0.98; *p* = 0.05), and over 6.00 million KRW (OR = 0.45; 95% CI, 0.19–0.99; *p* = 0.05) and residential area were associated significantly with healthcare avoidance. However, among women (*n* = 522), in their 50 s (OR = 3.05; 95% CI, 1.27–7.30; *p* = 0.01) or older than 60 (OR = 2.90; 95% CI, 1.23–6.82; *p* = 0.01) significantly influenced their healthcare avoidance. Factors that made people vulnerable differed among gender groups. When we restricted the respondents to those with an underlying disease (*n* = 411), only the respondents’ residential area, Daegu/Gyeongbuk-region (OR = 4.26; 95% CI, 1.45–12.51; *p* = 0.01), was significantly related to their healthcare utilization. Among the respondents with no underlying disease, the following groups were more likely to avoid healthcare: females (OR = 2.02; 95% CI, 1.34–3.04; *p* < 0.001), those in their 30s (OR = 2.52; 95% CI, 1.29–4.93; *p* = 0.01), families of two or more (OR = 1.92; 95% CI, 1.00–3.77; *p* = 0.05), those with young children in the home (OR = 2.00; 95% CI, 1.07–3.73; *p* = 0.03), and those living in Daejeon/Sejong/Chungcheong-do (OR = 2.14; 95% CI, 1.00–4.62; *p* = 0.05) and Daegu/Gyeongbuk (OR = 2.51; 95% CI, 1.10–5.76; *p* = 0.03).

## 4. Discussion

Our findings provide useful insights for understanding the under-utilization of healthcare services in terms of demand by investigating the avoidance of healthcare associated with the COVID-19 pandemic, an emerging infectious disease. Among respondents, 73.2% avoided healthcare utilization, while only 26.8% did not. There was no statistically significant difference in the prevalence of healthcare avoidance between those with (72.0%) and without (74.9%) an underlying disease. The results indicate that the general population avoided visiting health facilities as a response to the COVID-19 outbreak, regardless of whether public health authorities recommended that they do so. We also identified sociodemographic factors (i.e., gender, age, income level, residential area) influencing the avoidance of healthcare utilization. The present study shows that not all societal groups share the burden of healthcare avoidance equally, as it disproportionately affects those with certain sociodemographic characteristics.

A few interesting findings should be highlighted. First, avoiding hospitals was prominent during the peak of the COVID-19 outbreak, which can potentially damage the overall health of the population and disrupt daily life. During the outbreak, the Korean government and public health authorities had not given any public health advice about postponing or avoiding visits to hospitals. Instead, officials made efforts to ensure access to safe and reliable care by encouraging the public to utilize healthcare when needed. The Korean government has designated a “National Relief Hospital,” that operates a screening clinic to separate potential COVID-19 infected patients and treats patients with respiratory infections in a separate place. Moreover, the transmission of the COVID-19 virus mostly occurred by community-acquired infection, not in hospitals.

Widespread healthcare avoidance might relate to the South Koreans’ experience with the Middle East Respiratory Syndrome (MERS) in 2015, as the COVID-19 outbreak brings back memories of MERS. Between the first documented occurrence of MERS infection (20 May 2015) and diagnosis of the last case (4 July 2015), there were 186 confirmed cases, with 38 deaths and 16,752 people quarantined [26]. All confirmed cases of MERS were suspected to be hospital-acquired infections except for one case of household transmission, and hospital-to-hospital transmission occurred in 17 hospitals, all of which originated in one hospital [26]. Avoiding hospitals even when sick during the 2015 South Korean MERS outbreak may have been a strategy for reducing the perceived risk of infection, as most MERS infections occurred at hospitals; the uncertainty about viral spread was very high. However, unlike the MERS virus, the spread of the COVID-19 virus has occurred primarily in communities. Although there is a distinct difference between the two viruses, the public might fear a nosocomial infection, and hold other misconceptions about the virus. This should be investigated further.

Second, socio-demographic characteristics (i.e., gender, age, income level) and especially residential area, were highly related to healthcare avoidance. Women, older people, those with a lower income level, and those living in highly affected residential areas were more likely to avoid healthcare utilization than other groups were. These results are similar to prior research investigating the association between social determinants and healthcare avoidance during public health emergencies such as epidemic outbreaks [18,23,27]. Therefore, the avoidance of behaviors of subpopulation members during a pandemic warrant the attention of health policy officers and public health authorities. Especially, elderly people in need of care need the support of family and friends or caregivers [27].

Among the investigated influencing factors, residential area had the most significant effect on healthcare avoidance. In particular, living in Daegu or Gyeongbuk (North Gyeongsang Province) regions, where COVID-19-confirmed patients exploded at the time of this study, have been found to be the strongest influencing factor in avoiding hospital visits. For example, among men, respondents living in the Daegu and Gyeoungbuk region were 4.87 times more likely to avoid healthcare than those living in Seoul. In the peak of the outbreak, the daily new patient count in Daegu had reached 741 by February 29, and thousands waited for hospital beds as cases surged [28]. At the time of this study, cumulative cases in Daegu had reached 6456 (25 March). One can reasonably expect that citizens of Daegu/Gyeongbuk were at increased risk due to healthcare under-utilization during the COVID-19 outbreak. Fortunately, many medical staff and volunteers both local and from all over the country have come and participated voluntarily to help overcome the crisis in Daegu [15].

There are a number of implications that have emerged from this study. First, health authorities must make efforts to sustain the efficacy of the healthcare systems by providing sufficient support for the public to utilize proper healthcare services on both the demand-side and the supply-side. For the demand-side, instructions on how and when to visit the hospital should be provided to patients with non-infectious diseases in order to prevent inappropriate healthcare avoidance. While controlling the spread of infectious disease quickly is the urgent primary goal of the public health authorities, guidelines for people in terms of maintaining their health is also very important [29]. At the same time, standards and procedures should be prepared to treat non-infected patients in all possible clinical situations. On the supply-side, human resources of medical experts, experts in public health and epidemics, along with new policies are needed to improve the resilience of highly affected communities. Second, it is expected that the number of patients visiting hospitals has drastically decreased, causing financial losses in the healthcare facilities. Negative financial impacts of outbreaks have been reported in previous studies [30,31]. Various support plans should be prepared, including financial arrangements to compensate for the loss of medical institutions.

Our study has several limitations. First, the analyses did not extensively explore psychological factors such as the perceived risk or fear of visiting hospitals and trust in public health authorities. Therefore, we did not investigate the psychological factors influencing healthcare avoidance, so further research is needed. Second, we could not identify whether healthcare avoidance resulted from misconceptions about the spread of COVID-19, which some might perceive as a nosocomial infection. Future studies should measure and analyze knowledge of the virus as an independent variable. Third, this study is based on questionnaires which investigated the self-reported healthcare service avoidance. Moreover, this study design is cross-sectional and is not available to examine the trend of healthcare avoidance during the pandemic. Further research using national data, such as Korea National Health Insurance (KNHI) Claims Database, would be able to investigate actual numbers of healthcare utilization and change over time during the pandemic. Finally, this study did not investigate the avoidance of healthcare service for reasons other than COVID-19, which can confound the findings of this study.

## 5. Conclusions

In conclusion, the results of this study documented that a noticeable proportion of the public avoided healthcare visits who under-utilized healthcare resources that had not been advised by the government during the COVID-19 outbreak. Subgroups who were more likely to avoid visiting hospitals were identified, with residential areas playing a significant role in respondents’ behaviors. This study offers guidance for developing public health policy making to establish customized healthcare utilization policies and health promotion for specific groups of individuals. Prioritizing policies and efforts will be necessary for these vulnerable populations to reduce unmet healthcare needs. Understanding the patterns of healthcare utilization during infectious disease outbreaks would be valuable for facilitating appropriate responses and reducing the negative impact on population health.

## Figures and Tables

**Table 1:** General characteristics of the study participants.

Characteristics	Total (*n* = 1000)	
Socio-Demographics	*n*	%
Gender		
Male	478	47.8
Female	522	52.2
Age (year)	M = 47.04	SD = 15.04
18–29	165	16.5
30–39	157	15.7
40–49	197	19.7
50–59	205	20.5
≥60	276	27.6
Family size, No.		
1(living alone)	99	9.9
more than 2	901	90.1
Education level		
Middle school or below	29	2.9
High school graduate	481	48.1
College and above	490	49.0
Marital status		
Married	649	64.9
Single/divorced/bereaved	351	35.1
Presence of children		
None	903	90.3
More than 1	97	9.7
Monthly household income		
Under 200	129	12.9
200–400	315	31.5
400–600	262	26.2
≥600	294	29.4
Residence		
Urban	880	88.0
Rural	120	12.0
Residential areas		
Seoul	193	19.3
Incheon/Gyeonggi	308	30.8
Daejeon/Sejong/Chungcheong	105	10.5
Gwangju/Jeolla	95	9.5
Daegu/Gyeongbuk	99	9.9
Busan/Ulsan/Gyeongnam	159	15.9
Gangwon/Jeju	41	4.1
Occupation status		
Salary earner	473	47.3
Self-employed	131	13.1
Out of labor	396	39.6
Health-related factors	*n*	%
Subjective health		
Bad	116	11.6
Moderate	442	44.2
Good	442	44.2
Underlying disease		
None	589	58.9
More than 1	411	41.1
Avoidance of healthcare utilization	*n*	%
Never	268	26.8%
Sometimes	266	26.6%
Often	223	22.3%
Always	243	24.3%

**Table 2:** Chi-square statistics for variables related to healthcare utilization avoidance.

Variables	Sample Size (*n*)	Avoid Healthcare Utilization		
		“Never”	“Otherwise”	*p*-Value
*Socio-demographics*				
Gender				<0.001
Male	478	156 (32.6%)	322 (67.4%)	
Female	522	112 (21.5%)	410 (78.5%)	
Age				<0.001
18–29	165	63 (38.2%)	102 (61.8%)	
30–39	157	34 (21.7%)	123 (78.3%)	
40–49	197	56 (28.4%)	141 (71.6%)	
50–59	205	46 (22.4%)	159 (77.6%)	
≥60	276	69 (25.0%)	207 (75.0%)	
Family size, No.				0.29
1(living alone)	99	31 (31.3%)	68 (68.7%)	
more than 2	901	237 (26.3%)	664 (73.7%)	
Education level				0.38
Middle school or below	29	7 (24.1%)	22 (75.9%)	
High school graduate	481	120 (24.9%)	361 (75.1%)	
College and above	490	141 (28.8%)	349 (71.2%)	
Marital status				0.02
Married	649	158 (24.3%)	491 (75.7%)	
Single/divorced/bereaved	351	110 (31.3%)	241 (68.7%)	
Presence of children				0.15
None	903	248 (27.5%)	655 (72.5%)	
More than 1	97	20 (20.6%)	77 (79.4%)	
Monthly household income				0.12
Under 200	129	32 (24.8%)	97 (75.2%)	
200–400	315	71 (22.5%)	244 (77.5%)	
400–600	262	75 (28.6%)	187 (71.4%)	
≥600	294	90 (30.6%)	204 (69.4%)	
Residence				0.53
Urban	880	233 (26.5%)	647 (73.5%)	
Rural	120	35 (29.2%)	85 (70.8%)	
Residential area				0.01
Seoul	193	66 (34.2%)	127 (65.8%)	
Incheon/Gyeonggi	308	86 (27.9%)	222 (72.1%)	
Daejeon/Sejong/Chungcheong	105	23 (21.9%)	82 (78.1%)	
Gwangju/Jeolla	95	25 (26.3%)	70 (73.7%)	
Daegu/Gyeongbuk	99	15 (15.2%)	84 (84.8%)	
Busan/Ulsan/Gyeongnam	159	46 (28.9%)	113 (71.1%)	
Gangwon/Jeju	41	7 (17.1%)	34 (82.9%)	
Occupation status				0.56
Salary earner	473	122 (25.8%)	351 (74.2%)	
Self-employed or other job	131	40 (30.5%)	91 (69.5%)	
Out of labor	396	106 (26.8%)	290 (73.2%)	
*Health-related factors*				
Subjective health				0.08
Bad	116	27 (23.3%)	89 (76.7%)	
Moderate	442	107 (24.2%)	335 (75.8%)	
Good	442	134 (30.3%)	308 (69.7%)	
Underlying disease				0.30
None	589	165 (28.0%)	424 (72.0%)	
More than 1	411	103 (25.1%)	308 (74.9%)	

**Table 3:** Influencing factors associated with healthcare utilization avoidance (*n* = 1000).

Variables	Unadjusted		Adjusted	
	OR (95%CI)	*p*-Value	Adjusted OR (95%CI)	*p*-Value
*Socio-demographics*				
Gender				
Male	Ref.		Ref.	
Female	1.79 (1.34–2.38)	<0.001	1.91 (1.40–2.62)	<0.001
Age (year)				
18–29	Ref.		Ref.	
30–39	2.19 (1.33–3.60)	<0.001	1.85 (1.05–3.27)	0.03
40–49	1.53 (0.98–2.38)	0.06	1.26 (0.72–2.21)	0.42
50–59	2.09 (1.32–3.31)	<0.001	1.93 (1.06–3.50)	0.03
≥60	1.85 (1.21–2.83)	<0.001	1.46 (0.82–2.60)	0.2
Family size, No.				
1(living alone)	Ref.		Ref.	
more than 2	1.25 (0.79–1.97)	0.34	1.46 (0.83–2.56)	0.19
Education level				
Under middle school	Ref.		Ref.	
High school graduate	1.04 (0.43–2.51)	0.93	1.06 (0.42–2.69)	0.9
College and above	0.82 (0.34–1.97)	0.66	1.06 (0.41–2.74)	0.9
Marital status				
Married	Ref.		Ref.	
Single/divorced/bereaved	0.71 (0.53–0.95)	0.02	0.91 (0.58–1.42)	0.66
Presence of children				
None	Ref.		Ref.	
More than 1	1.42 (0.85–2.37)	0.18	1.19 (0.66–2.15)	0.57
Household monthly income				
Under 200	Ref.		Ref.	
200–400	1.07 (0.66–1.74)	0.79	0.98 (0.58–1.68)	0.95
400–600	0.79 (0.49–1.29)	0.35	0.65 (0.37–1.15)	0.14
≥600	0.69 (0.43–1.12)	0.13	0.61 (0.35–1.08)	0.09
Residential area				
Urban	Ref.		Ref.	
Town	0.86 (0.57–1.31)	0.49	0.65 (0.41–0.99)	0.05
Residential area2				
Seoul	Ref.		Ref.	
Incheon/Gyeonggi-do	1.30 (0.88–1.91)	0.19	1.37 (0.92–2.06)	0.12
Daejeon/Sejong/Chungcheong-do	1.80 (1.04–3.12)	0.04	2.04 (1.14–3.65)	0.02
Gwangju/Jeolla-do	1.45 (0.83–2.52)	0.19	1.49 (0.84–2.63)	0.17
Daegu/Gyeongbuk region	2.75 (1.47–5.16)	<0.001	3.10 (1.62–5.94)	<0.001
Busan/Ulsan/Gyeongnam region	1.29 (0.82–2.05)	0.27	1.30 (0.81–2.09)	0.28
Gangwon/Jeju	2.38 (1.00–5.67)	0.05	2.78 (1.12–6.88)	0.03
Occupation status				
Salary earner	Ref.		Ref.	
Self-employed or other job	0.78 (0.51–1.20)	0.26	0.77 (0.50–1.21)	0.26
Out of labor	0.96 (0.71–1.31)	0.81	0.75 (0.52–1.08)	0.13
*Health-related factors*				
Subjective health				
Bad	Ref.		Ref.	
Moderate	0.98 (0.60–1.59)	0.93	1.01 (0.61–1.69)	0.96
Good	0.71 (0.44–1.15)	0.17	0.79 (0.47–1.34)	0.39
Underlying disease				
None	Ref.		Ref.	
More than 1	1.17 (0.87–1.56)	0.3	0.97 (0.69–1.38)	0.88

**Table 4:** Influencing factors associated with healthcare utilization avoidance among subgroup participants along with gender.

Variables	Adjusted OR (95%CI)	*p*-Value	Adjusted OR (95%CI)	*p*-Value
	Male Subgroup (*n* = 478)		Female Subgroup (*n* = 522)	
*Socio-demographics*				
Age (year)				
18–29	Ref.		Ref.	
30–39	1.71 (0.76–3.84)	0.19	2.01 (0.85–4.74)	0.11
40–49	0.83 (0.36–1.92)	0.67	1.73 (0.77–3.88)	0.18
50–59	1.08 (0.44–2.62)	0.87	3.05 (1.27–7.30)	0.01
≥60	0.66 (0.28–1.57)	0.34	2.90 (1.23–6.82)	0.01
Family size, No.				
1(living alone)	Ref.		Ref.	
2 or more	1.63 (0.74–3.58)	0.22	1.70 (0.71–4.11)	0.24
Education level				
Middle school or below	Ref.		Ref.	
High school graduate	3.15 (0.88–11.26)	0.08	0.30 (0.04–2.44)	0.26
College and above	2.89 (0.79–10.60)	0.11	0.34 (0.04–2.81)	0.32
Marital status				
Married	Ref.		Ref.	
Single/divorced/bereaved	0.58 (0.30–1.14)	0.12	1.25 (0.64–2.46)	0.51
Presence of children				
None	Ref.		Ref.	
More than 1	1.25 (0.49–3.19)	0.64	1.23 (0.55–2.74)	0.62
Household income/mo.				
Under 200	Ref.		Ref.	
200–400	0.91 (0.41–2.02)	0.81	0.85 (0.39–1.84)	0.68
400–600	0.43 (0.18–0.98)	0.05	0.74 (0.32–1.71)	0.48
≥600	0.45 (0.19–0.99)	0.05	0.62 (0.27–1.40)	0.25
Residence				
Urban	Ref.		Ref.	
Rural	0.53 (0.28–1.01)	0.05	0.87 (0.44–1.74)	0.70
Residential area				
Seoul	Ref.		Ref.	
Incheon/Gyeonggi	2.02 (1.14–3.58)	0.02	0.86 (0.47–1.58)	0.63
Daejeon/Sejong/Chungcheong	2.93 (1.30–6.57)	0.01	1.37 (0.57–3.28)	0.48
Gwangju/Jeolla	2.80 (1.23–6.36)	0.01	0.82 (0.35–1.89)	0.64
Daegu/Gyeongbuk	4.87 (1.93–12.28)	0.00	1.88 (0.73–4.87)	0.19
Busan/Ulsan/Gyeongnam	1.50 (0.77–2.91)	0.24	1.11 (0.54–2.31)	0.77
Gangwon/Jeju	4.97 (1.36–18.07)	0.02	1.69 (0.44–6.54)	0.45
Occupation status				
Salary earner	Ref.		Ref.	
Self-employed or other job	0.68 (0.37–1.24)	0.21	1.00 (0.48–2.10)	1.00
Out of labor	0.71 (0.40–1.27)	0.25	0.87 (0.52–1.44)	0.58
*Health-related factors*				
Subjective health				
Bad	Ref.		Ref.	
Moderate	1.44 (0.65–3.19)	0.36	0.76 (0.37–1.56)	0.46
Good	0.85 (0.38–1.88)	0.68	0.81 (0.39–1.68)	0.57
Underlying disease				
None	Ref.			
More than 1	0.90 (0.67–1.23)	0.88	0.78(0.54–1.05)	0.27

**Table 5:** Influencing factors associated with the avoidance of healthcare utilization among subgroup participants according to the presence of an underlying disease.

Variables	Adjusted OR (95%CI)	*p*-Value	Adjusted OR (95%CI) (95%CI)	*p*-Value
	With Underlying Disease (*n* = 411)		Without Underlying Disease (*n* = 589)	
*Socio-demographics*				
Gender				
Male	Ref.		Ref.	
Female	1.58 (0.94–2.65)	0.09	2.02 (1.34–3.04)	<0.001
Age (year)				
18–29	Ref.		Ref.	
30–39	0.69 (0.20–2.41)	0.56	2.52 (1.29–4.93)	0.01
40–49	1.12 (0.32–4.02)	0.86	1.22 (0.63–2.34)	0.56
50–59	1.63 (0.49–5.43)	0.43	1.87 (0.89–3.92)	0.10
≥60	1.11 (0.35–3.55)	0.86	1.54 (0.73–3.26)	0.26
Family size, No.				
1(living alone)	Ref.		Ref.	
more than 2	0.80 (0.25–2.54)	0.71	1.92 (1.00–3.77)	0.05
Education level				
Middle school or below	Ref.		Ref.	
High school graduate	1.33 (0.43–4.12)	0.62	1.18 (0.20–7.03)	0.86
College and above	0.85 (0.27–2.70)	0.78	1.49 (0.25–8.98)	0.66
Marital status				
Married	Ref.		Ref.	
Single/divorced/bereaved	0.94 (0.43–2.04)	0.87	0.92 (0.52–1.63)	0.77
Presence of children				
None	Ref.		Ref.	
More than 1	1.31 (0.42–4.14)	0.64	2.00 (1.07–3.73)	0.03
Monthly household income				
Under 200	Ref.		Ref.	
200–400	1.04 (0.47–2.34)	0.92	1.01 (0.49–2.09)	0.97
400–600	0.90 (0.37–2.22)	0.82	0.61 (0.29–1.29)	0.20
≥600	0.61 (0.26–1.48)	0.28	0.68 (0.32–1.44)	0.31
Residence				
Urban	Ref.		Ref.	
Rural	0.76 (0.37–1.59)	0.47	0.52 (0.28–0.96)	0.04
Residential area				
Seoul	Ref.		Ref.	
Incheon/Gyeonggi	1.41 (0.75–2.68)	0.29	1.27 (0.74–2.18)	0.38
Daejeon/Sejong/Chungcheong	2.01 (0.80–5.05)	0.14	2.14 (1.00–4.62)	0.05
Gwangju/Jeolla	1.94 (0.74–5.10)	0.18	1.37 (0.66–2.85)	0.40
Daegu/Gyeongbuk	4.26 (1.45–12.51)	0.01	2.51 (1.10–5.76)	0.03
Busan/Ulsan/Gyeongnam	1.56 (0.74–3.27)	0.24	1.11 (0.59–2.09)	0.76
Gangwon/Jeju	3.67 (0.75–18.01)	0.11	2.30 (0.72–7.36)	0.16
Occupation status				
Salary earner	Ref.		Ref.	
Self-employed or other job	0.83 (0.41–1.68)	0.61	0.69 (0.38–1.27)	0.24
Out of labor	0.96 (0.53–1.73)	0.88	0.67 (0.42–1.08)	0.10
*Health-related factors*				
Subjective health				
Bad	Ref.		Ref.	
Moderate	1.44 (0.77–2.67)	0.25	0.50 (0.16–1.58)	0.24
Good	1.00 (0.51–1.96)	0.99	0.42 (0.13–1.33)	0.14

## Data Availability

The datasets used and analyzed in the current study are available from the corresponding author on reasonable request.
